# Environment-Related and Body-Related Components of the Minimal Self

**DOI:** 10.3389/fpsyg.2021.712559

**Published:** 2021-11-11

**Authors:** Marvin Liesner, Wilfried Kunde

**Affiliations:** Department of Cognitive Psychology, Julius-Maximilians-Universität Würzburg, Würzburg, Germany

**Keywords:** active self, exteroception, ideomotor theory, interoception, minimal self, self-construction, sense of agency, sense of ownership

## Abstract

Perceptual changes that an agent produces by efferent activity can become part of the agent’s minimal self. Yet, in human agents, efferent activities produce perceptual changes in various sensory modalities and in various temporal and spatial proximities. Some of these changes occur at the “biological” body, and they are to some extent conveyed by “private” sensory signals, whereas other changes occur in the environment of that biological body and are conveyed by “public” sensory signals. We discuss commonalties and differences of these signals for generating selfhood. We argue that despite considerable functional overlap of these sensory signals in generating self-experience, there are reasons to tell them apart in theorizing and empirical research about development of the self.

## Introduction

Which type of systems, biological or artificial, might develop a self? According to sensorimotor approaches of the self, only agents can do so (Gallagher, 2000; Verschoor and Hommel, 2017). Agents are systems that process sensory data and generate efferent activity, which changes these sensory data. In other words, agents are systems that act and perceive. A developing human can become an agent, so as a (simulated) robot can possibly do (Hafner et al., 2020). In humans, perception relates to all kinds of sensory data, which come across as different perceptual modalities (like vision, audition, proprioception, etc.), while efferent activity is generated by muscle contractions. Robots perceive and act depending on their sensory and motor equipment.

Agents develop a “minimal self” (Gallagher, 2000), provided two learning processes take place. First, agents sense that they causally change perceptual states by efferent activity. In humans, this perceived causality between one’s voluntary action and a perceived outcome is called a “sense of agency,” which can be measured in various ways (Haggard, 2017). For a robot to develop a minimal self, a corresponding representation of this causal knowledge is required (Hafner et al., 2020). Second, the agent has to sense that there is a spatially extended part of the perceptual world that is somehow “unique” in that it certainly identifies the agent as the source of a perceptual experience. This unique part of the world is the agent’s “body” (Gallagher, 2000). In humans, this experience is called sense of body “ownership,” and like sense of agency, it can be assessed in various ways including explicit and implicit measures (Tsakiris, 2010, 2017). In robots, the development of a corresponding representation of the physical extension of the robot might be construed as a sense of body ownership as well (Hoffmann et al., 2018; Zenha et al., 2018).

In this article, we discuss reasons to tell apart two types of perceptual events, body-related and environment-related (exteroceptive) events. Moreover, we argue that body-related events should be ascribed a special role when it comes to develop a minimal self. Body-related events include, but are not restricted to, sensory events, which are often subsumed under the term interoception. Interoception is often meant to describe various kinds of sensory signals that originate from biological bodies, including visceral signals such as heart rate and body temperature (Craig, 2009; Tsakiris, 2017). While we acknowledge the role of such visceral signals for self-development (cf., Gentsch and Schütz-Bosbach, 2015; Marshall et al., 2018), we use the term interoception to refer to body-related signals that originate from moving the body, such as proprioception and tactile perception. We are aware that this does not quite match the common definition of interoception. However, in lack of a better and more specific term to subsume proprioception and tactile perception and to avoid the clumsiness and missing precision of speaking of “body-related signals” throughout the manuscript, we will use the term interoception in the following to summarize these sensory signals. Exteroceptive perception on the other hand relates to sensory processing that has the potential to capture events that are distinct from the agent, such as vision or audition.

Why is it worth discussing, or better reminding of, reasons to distinguish between these interoceptive and exteroceptive events? We think there are two reasons for doing so. First, recent sensorimotor approaches to the self tend to treat them as more or less equivalent (Ma and Hommel, 2015; Verschoor and Hommel, 2017). According to sensorimotor approaches, control over perceptual events is sufficient to integrate these events into the self in terms of sense of agency, and subsequently, the sense of body ownership. For example, Ma and Hommel (2015) conclude “people perceive as their body everything that expresses their intentions, including things within reach that move ‘as they wish’ ” (p. 85). While we are generally sympathetic to this view, we want to highlight that control over exteroceptive events is important, but not sufficient, to induce body ownership experience. To experience ownership of exteroceptive events, control over these events must be accompanied by concurrent control over interoceptive events. Even with such concurrent control, a sense of body ownership of exteroceptive events sometimes fails to occur. For example, controlling an external object like a rubber hand does not come with an ownership experience of that rubber hand if it is placed in an anatomically implausible position (Kalckert and Ehrsson, 2012). Also, controlling a tool does not come with an ownership experience of the tool if it moves spatially incompatible to the operating hand (Liesner et al., 2020b). Second, to consider the role of interoceptive events might be particularly relevant when it comes to model how artificial agents, like robots, may or may not develop a self. If there is a special role in interoceptive events in human agents when it comes to self-development, as we believe, this raises the question in which way robots develop a self, similar to that humans have. Or, put differently: If one has the aim to develop a self in robots with sufficient similarity to the human self, how can one then account for this special role of interoceptive events? We want to make our case by assessing the role of interoceptive and exteroceptive events for two key components of a minimal self, the sense of agency and the sense of ownership. We then conclude by discussing some avenues for future research.

## Sense of Agency

What does it take to develop a sense of agency? Empirical research has shown that a match is important between perceptual changes the agent aimed at prior to generating efferent activity (often called “goals”), and the actual perceptual feedback after the efferent activity had been emitted (for a recent overview, refer Haggard, 2017). If there is a match between goal and feedback, it is likely that this feedback was caused by the agent’s efferent activity (Carruthers, 2012; Haggard and Chambon, 2012; Gallagher, 2013; Zaadnoordijk et al., 2019). If there is a mismatch, it is more likely that the postaction percept was caused by something else than efferent activity. In case of repeated matches between anticipation and perceptual feedback, this feedback can said to be controllable.

Models of motor control vary regarding the functional role that anticipation of feedback has. Some models construe such anticipations as “predictions” and the corresponding mismatch with actual perceptual feedback as a “prediction error” (Miall and Wolpert, 1996; Wolpert, 1997; Wolpert and Flanagan, 2001). These models thus assume that predictions are derived from the already specified efferent activity. We tend to favor a different view, which is called ideomotor control (James, 1981; Koch et al., 2004; Shin et al., 2010; Waszak et al., 2012; Hommel, 2013). This model assumes that the agents first accidentally produce efferent activity (motor babbling) and link the consistently ensuing perceptual changes to that efferent activity. Only after such links have been established, can an efferent activity be deliberately generated by recollecting the consistently produced perceptual changes, which are then named “goals.” Briefly, efferent activity is accessed through perceptual goals.

In this ideomotor model, prediction does not have a strong role. The perceptual goal is the best prediction that agents can possibly have about the outcome of their efferent activity, which is the very reason for activating this specific motor activity. Without these perceptual goals, they could not move intentionally at all. There is ample evidence suggesting that motor activities are indeed generated by recollecting their associated, and currently intended, perceptual changes (e.g., Elsner and Hommel, 2001; Kunde, 2001; Liesner et al., 2020a). To illustrate the difference between the two approaches, consider an example of a simple grasping action: Prediction-based models would assume that having the intention to achieve a certain end state of the action (i.e., grasping the object) triggers the implementation of a motor plan to achieve this intention. Based on this motor plan, a perceptual prediction is derived of how it should “feel” to achieve the intended end state of grasping the object, a so-called “efference copy.” The actual sensation while grasping is then compared with this predicted state (Miall and Wolpert, 1996; Wolpert, 1997; Wolpert and Flanagan, 2001). Ideomotor models, however, assume that the intended end state is essentially already an anticipation of the sensory consequences of the action. According to ideomotor models, one would thus anticipate how it “feels” to grasp the object and this anticipation would then trigger the necessary motor activities to achieve this sensory state. This link between motor activities and sensory effects is based on one’s learning history, which specific motor activities (e.g., grasping movements) and sensory effects (e.g., grasping sensations) have frequently occurred together (James, 1981; Koch et al., 2004; Shin et al., 2010; Waszak et al., 2012; Hommel, 2013). A sense of agency would then be inferred from the match between intended effects (i.e., goals) and actually observed effects. We tend to favor this in our view more parsimonious ideomotor approach over the prediction-based approach. We think that adding a further sensory prediction to the action planning phase when the intended sensory state is already known does not provide much benefit for the agent, and seems dispensable to explain agency and ownership experiences. We do not have the space here to discuss possible distinctions between “predictions” and “goals” further. For the purpose of the present paper, a strict differentiation of the two is not necessary since the model we want to propose in this paper only suggests that sensory anticipations of some form are made when engaging in the voluntary efferent activity. Moreover, different views on predictions and goals are actually not that incommensurate. Recent approaches suggest that predictions are the “motor commands” that generate efferent activity according to ideomotor theory (Brown et al., 2013; for a discussion of predictions versus goals see Dogge et al., 2019).

Here comes an important point. Depending on the sensory equipment of the agent, efferent activities typically produce all kinds of sensory feedback. Similarly, agents can have all kinds of perceptual goals. They might generate the same efferent activity at a superficial level to reach these different goals (Kunde and Weigelt, 2005; Pfister, 2019; Mocke et al., 2020). Think of a person controlling a tool such as a mouse cursor on a PC screen. After some experience with the tool (i.e., after associations between muscle contractions and cursor movements have been established), an agent might generate a movement of the tool by recollecting the visual tool trajectory (i.e., anticipating the cursor movements on the screen). Yet, the agent might also produce the superficially same movement by recollecting the proprioceptive sensations of the corresponding hand movement. There is in fact evidence that agents prefer either one or the other type of perceptual goal, depending on certain factors such as the spatial match between visual and proprioceptive feedback of the motor pattern and the specific task demands (Heuer and Rapp, 2012; Liesner and Kunde, 2020). Is there room for a special role of interoceptive (e.g., proprioceptive) compared with exteroceptive (e.g., visual) motor feedback? Not really. Perhaps the only special role of interoception is that, due to lifelong experience, starting before birth, human agents amass conceivably closer links between efferent activities and interoceptive feedback than they do with any possible exteroceptive feedback. But that is just a gradual rather than a qualitative difference.

But does this mean that every controllable perceptual state becomes part of the self, so as sensorimotor approaches to the self suggest (Ma and Hommel, 2015; Verschoor and Hommel, 2017)? There are both empirical findings and logical arguments that suggest that this is not the case. For example, studies investigating different measures of the sense of agency found that participants experienced less agency when efferent activities led to spatially discrepant interoceptive and exteroceptive signals than when there was no such discrepancy, despite equal controllability (Ebert and Wegner, 2010; Liesner et al., 2020a). According to ideomotor theory, this effect is due to the links of discrepant signals with different, conflicting motor patterns. Most importantly, however, these results are only explainable when keeping up a conceptual differentiation between interoceptive and exteroceptive effects of efferent activities. It has been shown that similar discrepancies between exteroceptive effects only do not lead to such a reduction in the sense of agency (Grechuta et al., 2019). Furthermore, if it would just be controllability of sensory input that determines what we call self, essentially everything we see was part of our self: If we move the eyes to the left, everything on the retina moves to the left. Therefore, every visual object a human can perceptually manipulate by moving the eyes (essentially every visual object) would be part of the self. While this motor-sensory contingency is for sure important to develop consciousness (O’Regan and Noë, 2001; O’Regan, 2011), not every stimulation that reaches consciousness is construed as being part of the self. Also, if it would just be controllability of perceived objects, which determines inclusion of these objects to the self, an agent could not tell apart a mirror image of the agent from the agent. This is sometimes portrayed in a slightly simplified manner in research of self-development in robots. A robot might well detect that it controls a visual mirror image (Hoffmann et al., 2021), but that does not mean that it has developed a self. By contrast, human agents and many animals, starting from a certain age on, can distinguish their “body” from a mirror image of their body (e.g., Gallup, 1970; Amsterdam, 1972; Reiss and Marino, 2001). But how can they do so?

## Sense of Body Ownership

In humans, and perhaps other biological agents, the likely answer to this question is: Because there are unique perceptual events, processed by specific neuronal pathways and cortical regions like the insular, anterior cingulate, or somatosensory cortex (Critchley et al., 2004; Craig, 2009), which can be summarized under the heading interoception. In the context of self-development, the term “interoception” might be a bit misleading, because it suggests that there was already something “interior” (inside the body) and something “exterior” (outside the body), which is the very distinction that the system has to develop in the first place. The crucial point is, however, that there is one, and only one, and thus unique object in the world that can generate “interoceptive” perception, the object that human agents call their “body.” For example, we can see that an object touches another object or another agent, so as we can see that an object touches the hand. Yet, only the hand generates the specific perceptual experience of being touched. In psychological theorizing around the concepts of mirroring or empathy, it is sometimes suggested that observers could directly perceive “feelings” or internal states of an observed other agent (e.g., Singer and Lamm, 2009). No, they cannot. The agents might directly see or hear another agent moving, so as they can directly see or hear themselves moving. But only indirectly, by matching that visual or auditory experience to corresponding interoceptive sensations, including those that originate from own moving limbs, the agents might ascribe interoceptive states to another agent (Rizzolatti, 2005; Schütz-Bosbach and Prinz, 2007). Therefore, the agents also cannot mirror the “feeling” of another observed agent, if they have no sufficient recollection of experiencing this “feeling” before themselves (Bosbach et al., 2005). Moreover, the agents cannot imitate other agents, without establishing a linkage between exteroception and interoception through observation of own motor activities. Reports of “imitation” in newborns without that correspondence experience have been criticized on empirical grounds (Slaughter, 2021), or as being expressions of an innate stimulus-response link, where the seen action of a model accidentally matches the innate response of the imitator when judged from a third party (Heyes, 2001). A similar argument has been put forward by phenomenological philosophers in the context of the so-called “analogy argument.” This argument suggests that the agents only have access to the internal states of other agents by inferring these from observing the other agents’ external states and drawing conclusions based on their own experiences with typical combinations of internal and external states within themselves (Husserl, 1973; Zahavi, 2001). Some authors have even suggested that only because of one’s experience with own interoceptive and exteroceptive sensations accompanying each other, one can also understand the existence of others and their selves as entities that are different from one(’s)self (Merleau-Ponty, 1945, 1964; Husserl, 1959). Differentiating between interoceptive and exteroceptive signals would thus not only be essential for developing a sense of self, but also for recognizing other agents, which is a crucial skill in the inherently social world that we as humans live in.

It should be noted that the relevant aspect of interoceptive sensory signals for selfhood experiences is not their sensory modalities *per se*, but rather that they diagnostically and infallibly signal the presence of an agent’s physical body. In healthy human agents, this function is taken by interoceptive signals, however, in principle, this function could also be taken by other signals, given that they are “exclusive” enough for providing information about the agent’s body. We will discuss this possibility further in the context of artificial agents and patients suffering from deafferentation (see next paragraph and section “Agents Without Interoceptive Perception”).

Put differently, some perceptual effects of motor activities like visual effects are “public.” An agent perceives them more or less, so as other agents do. No doubt, this “publicity” is very important, as it allows matching activities of different agents to each other, and ascribing internal states to other agents, among other things. However, to ascribe uniqueness to an agent’s body, controllable sensory events that do arise from just this unique object (the “body”) and which are apparent to just the agent, are certainly helpful, if not mandatory. As only the agent has these unique experiences, these experiences might be called “private” (i.e., reserved to the observing agent). In technical systems, these need not necessarily be proprioceptive or tactile events like in humans (if the comparison to human sensory systems makes sense at all). But there has to be some kind of perceptual event that no other object except the agent’s physical body can generate. Over the past years, some robotics studies have introduced methods that might be possible candidates for such “private” sensations (Nabeshima et al., 2005; Roncone et al., 2014; Hinz et al., 2018; Hoffmann et al., 2018; Lanillos and Cheng, 2018). For example, information read out from the joint positions of the robot have been suggested as a proxy to proprioceptive sensations (Nabeshima et al., 2005), while pressure sensors in an “artificial skin” on the robot have been used as a source for modeling tactile information (Hinz et al., 2018; Hoffmann et al., 2018). It is beyond the scope of this article to evaluate the adequacy of these approaches and whether they can “substitute” the function of interoceptive sensations in humans. The point that we want to make is that some “private” input of whatsoever form is a necessary prerequisite for the development of an (artificial) self.

The idea that interoceptive signals provide very diagnostic information about the presence of one’s (bodily) self has already been put forward by other authors when discussing the principle of “immunity to error through misidentification” (Cassam, 1995; Gallagher, 2013). These authors have suggested that, while sensory information that we would label as exteroceptive (e.g., visual) can be misleading regarding whether it stems from one’s own body or not, proprioceptive (i.e., interoceptive) information necessarily signals the presence of one’s body since it cannot be perceived for anything or anybody else. As Gallagher (2013) explains, proprioceptive perception can still be erroneous in terms of, for example, the perceived position of a body part (see next paragraph), but there can be no erroneous experience of a perceived proprioceptive signal as not stemming from the own biological body. This view of the innate self-reference of proprioceptive signals is very much compatible with our argumentation that interoceptive signals take a special role regarding the formation of (body) ownership experiences. However, while the previous works mainly focused on the impossibility to misjudge interoceptive signals as not originating from one’s own body, we want to make the point that a sense of ownership cannot be experienced at all without any unique sensory experiences, like interoceptive sensations in humans.

As mentioned before, motor activities, at least in neurotypical agents, mostly produce public and private signals at the same time. We can see and feel our hand moving or being touched, and we make a repeated experience that these perceptual events normally coincide in space and time, such that we see and feel a hand moving rightward. Because interoceptive events, like touch, are very diagnostic for body ownership, but have a low spatial accuracy, human agents sometimes misjudge visual events as indicating body ownership, if these visual events temporally coincide with interoceptive percepts despite moderate spatial displacement to these corresponding interoceptive events. This is the functional basis behind the so-called rubber hand illusion and other body-transfer illusions (e.g., Slater et al., 2010; Tajadura-Jiménez et al., 2012; Maselli and Slater, 2013). In the original experiment by Botvinick and Cohen (1998), a rubber hand that is seen to be stroked while the own hand is felt being stroked appears as belonging to the body. Thus, the temporal coincidence of interoceptive and exteroceptive events can create the impression that exteroceptive events belong to the same entity that normally produces interoceptive events, the body. Importantly, this, however, does not contradict the conceptual differentiation between interoceptive and exteroceptive sensations that we want to make in this study. Even if exteroceptive events are integrated with interoceptive events and the source of the latter might thus be experienced as belonging to one’s body, this does not mean that the interoceptive and exteroceptive sensations are experienced any differently *per se*. In the rubber hand illusion, the stroking on one’s real hand is mislocalized on the artificial hand (e.g., Dummer et al., 2009; Rohde et al., 2011; Kalckert and Ehrsson, 2012). However, this does not qualitatively change the interoceptive, tactile sensation felt by the brushstroke in any way. Similarly, also the exteroceptive, visual sensations from the rubber hand are not experienced qualitatively differently. For example, the rubber hand does not look any different for a participant experiencing the rubber hand illusion from what it looks like without experience of the illusion (Botvinick and Cohen, 1998; Rohde et al., 2011). “Integration” of interoceptive and exteroceptive signals thus does not mean that a new “synthesized” percept is created: Instead, some features of the sensation in one modality are shifted toward features in the other modality, the size and direction of which are influenced by the reliability of the sensory signals (Ernst and Banks, 2002; Tsakiris, 2010, 2017; Blanke, 2012). Even in cases of such integration of interoceptive and exteroceptive sensations, a differentiation between them, like we have suggested in this article, still holds.

A coincidence of interoceptive and exteroceptive signals can also be actively generated by efferent activity, thus when moving a body limb that moves another artificial limb (“active rubber hand illusion,” Kalckert and Ehrsson, 2012), or another non-corporeal object (Ma and Hommel, 2015). While such body ownership illusions suggest surprising plasticity of what counts as body, they are constrained to cases where there is concurrent, actively produced, interoceptive stimulation. Recently, it has been shown that the mutual relationship of various exteroceptive feedback signals might shape ownership experience (Grechuta et al., 2019), however, also in this case, task-related, interoceptive signals were still present. We are not aware of cases in which the coincidence of, for example, the visual experience of a moving object and a corresponding auditory event alone create ownership experience for that object even close to the range that occurs when interoceptive stimulation is involved.

In biological agents, there is another reason to ascribe interoception a special role. Put simply, every point in space that generates the feeling of touch can bleed, while only few parts of the visual world can do so (those parts of the anatomical body that are visible). It is thus no wonder that biological agents keep an eye on their body, even when they control tools, that otherwise appear to be “embodied” (Collins et al., 2008). After all, on an even higher, reflective level of representation, the lack of some interoceptive stimulation that tools cannot provide is often the very reason for using tools. For example, we use sticks to broil sausages in a campfire rather than our hands. True, depending on the amount of barbecue experience, and corresponding (sense of) agency over the tool, such a tool might appear as being part of the anatomical body (Maravita and Iriki, 2004; Liesner et al., 2020b), but it cannot produce heat pain, which is why we use it. Conceivably, the experience of tool ownership, despite lack of heat perception, does occur only because the tool movements coincide with other interoceptively (i.e., proprioceptively) sensed movements of the operating hand. Additionally, interoceptive signals are not only important to keep the biological substrate of the agent from harm, but they are also essential for the homeostatic and allostatic regulation of the body and the brain (Sterling, 2004, 2012; Barrett et al., 2016; Burleson and Quigley, 2021).

Coincidence of actively generated interoceptive and exteroceptive stimulation is necessary but not sufficient to assign exteroceptive stimulation bodilyness. Specifically, if any object under an agent’s immediate control would be experienced by this agent as belonging to their self, the specific relationship of the interoceptive signals from the body controlling the object and of the exteroceptive signals from the object itself should be negligible. Yet, that relationship does count. The explicit and implicit measures of the sense of ownership are decreased or even eliminated when interoceptive or exteroceptive signals are not sufficiently overlapping in terms of direction, location, or timing (e.g., Samad et al., 2015; Pritchard et al., 2016; Kalckert et al., 2019).

To illustrate this point, consider a recent study by Liesner et al. (2020b). The participants were asked to move a visual cursor on a screen by moving their occluded hands. In one (compatible) condition, the cursor moved to the same extent and in the same direction as the felt hand, whereas it moved to the same extent but to the opposite direction as the felt hand, in another (incompatible) condition. At an objective level, controllability of the cursor was identical in both conditions, that is, it was perfectly foreseeable how the cursor would move when then hand moved in both cases. While there were clear indications of ownership experience in the compatible condition, there was no indication of such ownership experience in the incompatible condition. Why is this so? We conjecture that the agents suppress interoceptive codes of their body movements in the incompatible conditions, as these codes cause interference during action planning (Janczyk and Kunde, 2020), a phenomenon also known as “haptic neglect” (Heuer and Rapp, 2012). Because of this suppression of interoceptive codes, it becomes much harder, or even impossible, to establish the coincidence of exteroceptive and interoceptive codes that is crucial to induce a sense of body ownership for exteroceptive events. Thus, exteroceptive codes are not integrated indiscriminately into the self, but only when they match with sufficiently strong interoceptive codes.

## Interim Summary and Future Directions

**FIGURE 1:**
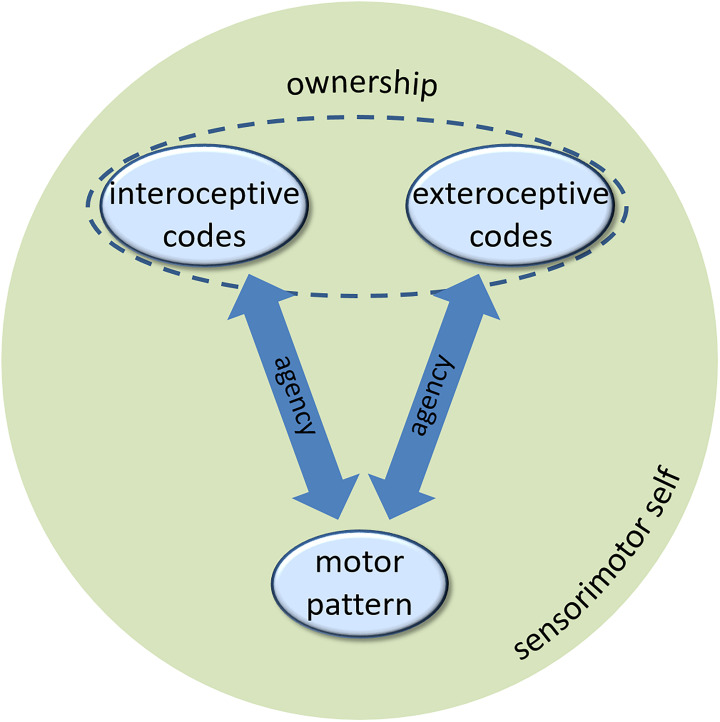
The role of interoceptive and exteroceptive codes in generating the sense of agency and the sense of ownership of a minimal sensorimotor self (see text for description).

Let us briefly summarize. The agents develop a sense of agency, a key component of a minimal self, based on controllable perceptual feedback of their efferent activity. The perceptual feedback in humans comes in various modalities, but there seems no fundamental reason to ascribe interoceptive feedback a special role. The agents can experience agency for interoceptive and exteroceptive events, in the same way, varying, if at all, gradually depending on the strength of associations to the motor patterns that cause these events (cf., Figure 1). Yet, there is conceptual and empirical reason to assume that another component of the self, the sense of body ownership, presumes perceptual feedback that no other part of the environment provides. In biological agents, this uniqueness applies to interoceptive sensory signals. In artificial agents, some other conceptualization of this feedback might be possible, given it provides the artificial agent with the same information about the artificial “body” as interoceptive information does for the biological body in biological agents. The sense of ownership of exteroceptive events rests on their integration with interoceptive codes. At the same time, interfering interoceptive and exteroceptive codes of the same action seem to lead to a suppression of the former (Fourneret and Jeannerod, 1998; Knoblich and Kircher, 2004; Müsseler and Sutter, 2009; Sülzenbrück and Heuer, 2009; Heuer and Rapp, 2012; Liesner and Kunde, 2020). While we have demonstrated that ownership experience of exteroceptive events is hard to acquire in these situations, it seems plausible that the unavailability of interoceptive codes either because of haptic neglect or due to loss of neural pathways (as in deafferented patients), might be the causal reason for this. This causal relationship is however, yet to be shown in empirical research. Taking this assessment for granted for a moment, a couple of research questions arise, which we discuss in the following.

### Developmental Order of the Sense of Ownership and the Sense of Agency

In the original rubber hand illusion, the ownership experience is induced by “passive” tactile-visual stimulation. This suggests that ownership experience might arise before, or even without, the agent has learned to move in a goal-oriented manner, and hence the experience of agency. However, it is not too far-fetched to assume the exact opposite order of development. As Hinton (2007, p. 535) has put it, “To recognize shapes, first learn to generate images.” In other words, to appropriately encode stimulation, observers have to first create that stimulation on purpose. In fact, the interpretation of touch is tightly coupled to active exploration, thus haptics (Lederman and Klatzky, 2009; Bremner and Spence, 2017). Nava et al. (2018) showed that actively applying stroking to oneself in the rubber hand illusion as compared to passively observing an experimenter stroking boosts the illusion in 5-year-old children, while this manipulation is known to rather lead to the opposite effects in adults. Particularly important, and extensively practiced by young infants is double touch, hence, touching the own “body,” which creates a tactile experience at both the touching and touched at body part, in the same position in space (Merleau-Ponty, 1954). Perhaps a proper encoding of touch (like being stroked) as diagnostic information for body ownership presumes a sufficient amount of haptic experience, which presumes goal-oriented action, and thus the experience of agency.

### Agents Without Interoceptive Perception

If interoceptive perception is the key to derive a sense of ownership, the possible ownership experience of agents without such interoception is a very interesting case. In fact, “deafferented” patients who have lost most of such interoceptive perception, sometimes report having a body that is not that clearly circumscribed. Some report that they experience their body as a tool to affect the environment (Cole and Paillard, 1995). It seems possible that the unique sensory experience of the body that interoceptive perception provides in neurotypical humans becomes substituted by some other (originally exteroceptive) indications of uniqueness, such as the unique visual appearance of the own hands and arms from an ego perspective. However, as the previously mentioned perception of one’s body as a tool or other reports of deafferented patients about a disembodied “floating” feeling after the onset of their condition suggests (Cole and Paillard, 1995), this substitution takes time and continued effort to achieve and sustain. These and other alterations in self- and body-perception in deafferented patients (Gallagher and Cole, 1995; Renault et al., 2018) suggest that the innate uniqueness of interoceptive sensations for signaling the presence of one’s own body is very difficult, if not impossible, to reach and replace with originally exteroceptive signals.

Deafferented patients are also interesting to study regarding the development of agency experience. In neurotypical human agents, the experience of agency is determined by long-term and short-term links of body movements and visual movement feedback. In most cases throughout lifetime, visual and proprioceptive feedback we get from our body spatially match. If this long-term link is violated by altering visual feedback, such that for example, the visual feedback of a movement is inverted relative to the proprioceptive feedback, as in mirror drawing, agency experience of the visual movement drops (Ebert and Wegner, 2010; Liesner et al., 2020a, b). Moreover, such a violation of long-term sensorimotor experience by short-term alterations of visual feedback comes with considerable drops of performance (Müsseler et al., 2008; Müsseler and Skottke, 2011; Kunde et al., 2012). Interestingly, patients with loss of interoceptive perception do not consistently show such a drop in performance (Lajoie et al., 1992). It seems likely that they do not experience reduced agency either. This may depend, however, on the way the sense of agency is explored. While tactile perception normally shapes the experience of, for example, temporal binding (Haggard, 2017), which is often considered an unobtrusive measure of the sense of agency (Cao et al., 2020), it is conceivable that these patients would still distinguish between normal and mirror drawing in their subjective experience of agency, just like neurotypical agents do (Ebert and Wegner, 2010; Liesner et al., 2020a). However, this agency experience would then most likely be based on the (mis)match of visual feedback in the environment and unique visual body representations, instead of unique proprioceptive body representations. Because many “standard” robots today are not yet equipped with sophisticated “interoceptive” sensors, the study of the sense of ownership and the sense of agency in deafferented patients might be quite inspiring for roboticists who aim to develop machines that contain these cornerstones of selfhood.

### Prosthesis Ownership Experiences and Phantom Limbs

Another interesting domain to study the role of intero- and exteroception are patients with limb prosthesis and/or phantom limb experiences. While the former basically provides a situation of a “body” part without any interoceptive sensation, similar to deafferented patients, the latter can be described as a case of illusory interoception (based on previous experiences; Ramachandran, 1998) without a corresponding body part. Recent studies have shown that extended motor control and sensory feedback from using a prosthesis enhances experienced ownership of the prosthesis and reduces phantom limb experiences (Page et al., 2018), while ownership experience of prosthesis and phantom limb experience are negatively correlated (Bekrater-Bodmann et al., 2021). This inverse relationship between prosthesis ownership experience and phantom limb experience might result from the transfer of memories of previous interoceptive perception from the lost limb to the prosthesis. Indeed, both subjective reports of prosthesis users and brain imaging studies suggest that prostheses can phenomenologically and neurally “replace” lost limbs and that the degree to which this happens is related to the level of satisfaction with and acceptance of the prosthesis (Maruishi et al., 2004; Murray, 2004). It might be interesting for future studies and treatment methods to investigate such a causal protective mechanism of prosthesis ownership experiences against often painful phantom limb experiences.

## Summary

The “self” is a glamorous term in social sciences. However, boiling down what it takes for an organism to develop a “self” is challenging. Sensorimotor approaches of this problem suggest that perceptual changes that are controllable by efferent activity tend to become part of the self. This approach is fascinating because it suggests that almost every controllable perceptual event in the world, be it visual, auditory, or proprioceptive, can count as self. This is probably true for the experience of agency. Yet, when it comes to developing a sense of having a body (sense of body ownership), there is conceptual and empirical reason to distinguish between proprioceptive or tactile (i.e., interoceptive) events and other controllable perceptual events. Proprioceptive and tactile events are exceptionally diagnostic to determine which parts of the world belong to the agent and which do not, which is of obvious importance to avoid the physical threat to the agent’s biological substrate. Lacking control over or perception of such events comes with severe decrements of the body ownership experience. Moreover, while controlled visual or auditory events might as well be construed by the agent as being owned, this happens only when these events coincide in a spatial and temporal manner with corresponding proprioceptive or tactile changes. Given these empirical observations in human agents, constructing machines that lack interoceptive sensation, but still develop a sense of body ownership in a similar manner as humans do, is a challenge.

## Author Contributions

ML and WK contributed to the conceptualization of the article, literature search, and writing of both the original and re-edited versions of the manuscript. Both authors approved the submitted version.

## Conflict of Interest

The authors declare that the research was conducted in the absence of any commercial or financial relationships that could be construed as a potential conflict of interest.

## Publisher’s Note

All claims expressed in this article are solely those of the authors and do not necessarily represent those of their affiliated organizations, or those of the publisher, the editors and the reviewers. Any product that may be evaluated in this article, or claim that may be made by its manufacturer, is not guaranteed or endorsed by the publisher.
